# Comparing measurement properties of EQ-5D-Y-3L and EQ-5D-Y-5L in paediatric patients

**DOI:** 10.1186/s12955-021-01889-4

**Published:** 2021-11-15

**Authors:** Titi Sahidah Fitriana, Fredrick Dermawan Purba, Rina Rahmatika, Riski Muhaimin, Nur Melani Sari, Gouke Bonsel, Elly Stolk, Jan J. V. Busschbach

**Affiliations:** 1 Department of Psychiatry, Section Medical Psychology and Psychotherapy, Erasmus MC University Medical Center, Wytemaweg 80, 3015 CN Rotterdam, The Netherlands; 2 Faculty of Psychology, YARSI University, Jakarta, Indonesia; 3 Department of Developmental Psychology, Faculty of Psychology, Universitas Padjadjaran, Jatinangor, Indonesia; 4 Department of Child Health, Cipto Mangunkusumo Hospital, Jakarta, Indonesia; 5 Department of Child Health, Faculty of Medicine, Universitas Padjadjaran/Dr. Hasan Sadikin Hospital, Bandung, Indonesia; 6 The EuroQol Research Foundation, Rotterdam, The Netherlands; 7 Department of Public Health, Erasmus MC University Medical Center, Dr. Molewaterplein 40, 3015 GD Rotterdam, The Netherlands

**Keywords:** EQ-5D-Y-5L, EQ-5D-Y-3L paediatric patients, Psychometrics, Health-related quality of life

## Abstract

**Background:**

The adult versions EQ-5D-3L and EQ-5D-5L have been extensive compared. This is not the case for the EQ-5D youth versions. The study aim was to compare the measurement properties and responsiveness of EQ-5D-Y-3L and EQ-5D-Y-5L in paediatric patients.

**Methods:**

A sample of patients 8–16 years old with different diseases and a wide range of disease severity was asked to complete EQ-5D-Y-3L, EQ-5D-Y-5L, PedsQL Generic Core Scale, and selected, appropriate disease-specific instruments, three times. EQ-5D-Y-3L and EQ-5D-Y-5L were compared in terms of: feasibility, (re-)distribution properties, discriminatory power, convergent validity, test–retest reliability, and responsiveness.

**Results:**

286 participating patients suffered from one of the following diseases: major beta-thalassemia, haemophilia, acute lymphoblastic leukaemia, acute illness. Missing responses were comparable between versions of the EQ-5D-Y, suggesting comparable feasibility. The number of patients in the best health state (level profile 11111) was equal in both EQ-5D-Y versions. The projection of EQ-5D-Y-3L scores onto EQ-5D-Y-5L for all dimensions showed that the two additional levels in EQ-5D-Y-5L slightly improved the accuracy of patients in reporting their problems, especially if severe. Convergent validity with PedsQL and disease-specific measures showed that the two EQ-5D-Y versions performed about equally. Test–retest reliability (EQ-5D-Y-3L 0.78 vs EQ-5D-Y-5L 0.84), and sensitivity for detecting health changes, were both better in EQ-5D-Y-5L.

**Conclusions:**

Extending the number of levels did not give clear superiority to EQ-5D-Y-5L over EQ-5D-Y-3L based on the criteria assessed in this study. However, increasing the number of levels benefitted EQ-5D-Y performance in the measurement of moderate to severe problems and especially in longitudinal study designs.

**Supplementary Information:**

The online version contains supplementary material available at 10.1186/s12955-021-01889-4.

## Introduction

One of the most widely used health-related quality of life (HRQoL) instruments is EQ-5D: a generic questionnaire that can provide a single index to be used in the calculation of Quality Adjusted Life Years (QALYs) [1]. The instrument was initially designed to be used in adult populations aged 18 and over [2]. In 2009, the EQ-5D-Y instrument was introduced for young respondents. EQ-5D-Y has similar dimensions and the same ordinal scaling as the adult instrument, but the dimension headings, the wording of labels, and the layout are adapted for maximal comprehensibility in children [3]. Initially, the response scale consisted of 3 ordinal levels [1]. Psychometric properties of this EQ-5D-Y-3L have been reported in several countries, indicating that the questionnaire is a valid and reliable instrument [4–7]. However, as with the adult version of EQ-5D, EQ-5D-Y-3L has been criticized for its lack of scaling options and its overt ceiling effects [7, 8]. To overcome this limitation, a modified version of EQ-5D-Y was developed with 5 response levels, the ‘EQ-5D-Y-5L’ instrument. The feasibility of EQ-5D-Y-5L has been confirmed by initial pilot-testing in 4 different countries [9].

A series of independent studies convincingly showed the superior psychometric properties of EQ-5D-5L compared to the EQ-5D-3L adult version, at no additional burden to the respondent [10–14]. While it is tempting to assume this evidence from the adult versions extends to the youth versions, this is not self-evident. The 5L and 3L youth labels are not identical to their adult counterparts. In addition, the increased complexity of 5L can have more impact on young respondents, which may translate into lower reliability and undesirable heuristics in their response behaviour.

A few studies have compared the 3L and 5L youth versions of EQ-5D in China [15–18]. The generalizability of these findings is, however, limited, as respondents predominantly had minor severity issues or were not patients. Hence, we launched a head-to-head comparison of the EQ-5D-Y-3L and 5L versions in children with a wide spectre of diseases and ages, a longitudinal design, and a suitable sample size. We addressed the following psychometric performance measures: feasibility, (re-)distribution properties, convergent validity, test–retest reliability, and responsiveness.

## Methods

### Participants

Participants were recruited from 5 hospitals located in Jakarta and Bandung, Indonesia. We included children aged 8–16 years, who had a good command of Bahasa Indonesia. There were 4 diagnostic groups involved: children with major beta-thalassemia, severe-to-moderate haemophilia, Acute Lymphoblastic Leukaemia (AcLL), and with acute disease requiring immediate clinical treatment. The 4 illness types were chosen in order to secure a broad spectre of severity, notably including patients with severe health states. We set out to include patients who underwent medical treatment so that responsiveness to detecting health changes over time could be compared. Patients were included after they and their parents signed informed consent.

### Disease groups

Children with *major beta-thalassemia* have impaired production of haemoglobin [19], causing severe anaemia. They have been diagnosed in their early lives and subsequently have received routine blood transfusions. They were expected to have some HRQoL problems due to their illness and to demanding treatment [20, 21].

*Haemophilia* is an impairment of the blood-clotting process that results in repeated spontaneous bleeding in joints and muscles [22]. In Indonesia, haemophiliacs are treated only if bleeding occurs [23]. We included patients with severe/moderate levels who usually had 1 bleeding/month [24]. Common HRQoL problems in haemophilia are mobility restriction, pain due to the onset of bleeding, and all types of social disturbances due to self-protective behaviour [22, 25].

*Acute Lymphoblastic Leukaemia* (AcLL) is a malignancy of the bone marrow that arises from several cooperative genetic mutations, which together lead to altered blast (precursor) blood cell development [26]. We differentiated AcLL patients into inpatients or outpatients to adjust the data collection window to their health characteristics. The inpatients were more unstable than the outpatients, hence retest assessment was undertaken a day after the baseline. Whilst for the outpatients, retest assessment was held within a week of the baseline. We expected AcLL children to have problems in all dimensions of HRQoL, especially during treatment or after treatment failure [27–29].

The final group of patients comprised acutely-ill children. We defined acutely-ill children as those who were hospitalized for sudden illnesses such as dengue, typhus, diarrhoea, or injury. Due to sudden onset, we expected this group of children to have problems in all dimensions of their HRQoL, including extreme levels.

### Study design

Ethical approval was obtained from the Ethical Committee of the Indonesian Ministry of Health—National Institute of Health Research and the respective hospital review boards. By default, the children were encouraged to complete the questionnaires by themselves. Only when they expressed difficulty filling in the questionnaire due to their health did interviewers offer them minimal help by reading aloud and writing down their answers. There were two trained interviewers in charge of each interview, one for the child and one for the parents. Parents provided clinical data for the patients and filled in the proxy version of EQ-5D-Y. The EQ-5D-Y proxy report is discussed elsewhere. Patients received Rp 100.000 (equal to 6 euros) at each meeting.

Patients were asked to complete a set of paper-and-pencil HRQoL questionnaires, on 3 occasions: (1) at baseline (*t*_*baseline*_); (2) at retest time (*t*_*retest*_), assumed to be in the same condition as at baseline; and (3) after receiving medical treatment (*t*_*followup*_). Due to the different nature of the diseases and treatments received by the patients, the order and collection time between, *t*_*baseline,*_* t*_*retest*,_ and *t*_*followup*_ was customized to the patient’s condition and the treatment window appropriate to the disease. Apart from 1 explicit question on health changes experienced (at *t*_*retest*_ and *t*_*followup*_) the questionnaire was the same on all occasions. Additional file 1 demonstrates the data collection time frame for each patient group. All respondents scored the 5L version first, as a previous study had shown a tendency to avoid level 2 and 4 in 5L when responding to the 3L first [14].

### Instruments

#### EQ-5D-Y

EQ-5D-Y is a generic instrument with 5 dimensions: mobility (walking about), looking after myself, doing usual activities, having pain or discomfort, and feeling worried, sad, or unhappy. In the standard 3L version, the response format has 3 severity levels: no problems, some problems, and a lot of problems [3]. In EQ-5D-Y-5L, 5 ordinal levels are deployed: no problems, a little bit of problems, some problems, a lot of problems, and cannot/extreme problems. Higher scores indicate worse outcomes. In addition to the descriptive system, EQ-5D also contains a Visual Analogue Scale (VAS) where participant health today is measured on a range of 0 to 100. At the time the study was conducted, the ‘standard’ UK English Version of EQ-5D-Y-5L was not available. Hence, in close collaboration with the Version Management Committee of the EuroQol Group, we translated the 'in progress’ UK English version of EQ-5D-Y-5L into Bahasa Indonesia, following the translation protocol of the Group.

#### PedsQL™ 4.0 Generic Core Scales

PedsQL™ 4.0 Generic Core Scales (Copyright © 1998 JW Varni, Ph.D. All rights reserved.) is a self-report questionnaire that consists of 23 items divided into 4 dimensions: physical, emotional, social, and school [30, 31]. Scores on the latter 3 dimensions can be summed to measure the psychosocial health summary score. Five level responses (0 to 4, where 0 means ‘never a problem’) are reversed and linearly transformed to a 0–100 scale (0 = 100, 1 = 75, 2 = 0, 3 = 25, 4 = 0). Average scores per dimension are computed, where higher scores indicate better HRQoL.

#### PedsQL cancer module

The PedsQL Cancer Module is a disease-specific questionnaire designed to assess the impact of disease and treatment on the HRQoL of paediatric cancer patients. The questionnaire consists of 27 items divided into 8 domains: pain and hurt, nausea, procedural anxiety, treatment anxiety, worry, cognitive problems, perceived physical appearance, and communication [27, 32]. There are five level responses from 0 to 4 where 0 means ‘never a problem’.

#### TranQol

TranQol is a disease-specific quality of life instrument for patients with thalassemia major [33]. There are 36 items grouped into 4 domains: physical, emotional, family functioning, and school/career functioning. The response option ranges from 0 (never a problem) to 5 (always a problem). An unofficial translation into Bahasa Indonesia of TranQol exists [34]. To confirm translation quality, we cognitively debriefed 3 children aged 12–15 with thalassemia. Based on their inputs, difficult wordings were simplified.

#### Haemo-Qol

Haemo-Qol is a disease-specific QoL instrument for children with haemophilia [35, 36]. The short version consists of 35 items divided into 8 dimensions: physical health, feeling, attitude, family, friends, other people, dealing with haemophilia, sport and school, and treatment. The items are scored from 1 to 5 where 1 indicates ‘never a problem’. The higher the score the lower the level of QoL.

### Additional Questions at Retest and Follow-up

#### General State of Health

We included a direct question about any perceived health state change by asking: *“Overall, has there been any change in your health compared to the first time you saw us? Please report any change by selecting one of the following options”*. Seven options were offered: much worse, moderately worse, slightly worse, no change, slightly better, moderately better, and much better. The first 3 answers (much worse, moderately worse and slightly worse) were considered to reflect a clinically significant deterioration, the fourth answer (no change) was considered to reflect stability, and the last 3 answers (slightly better, moderately better and much better) were considered to reflect a clinically significant improvement [37, 38].

### Analysis

#### Feasibility and ceiling effect

Feasibility was assessed by calculating the number of missing values in each of the participants’ questionnaires. The data ceiling was calculated as the proportion of respondents classifying themselves as having ‘no problems’ (level 1) in any of the 5 dimensions.

#### Redistribution properties

Any level response given in EQ-5D-Y-3L was expected to be redistributed in a logical way to a level in EQ-5D-Y-5L. The language specificity in Indonesia created complexity in the translation of ‘some problems’ in EQ-5D-Y instruments, notably level 2 of the 3L and level 3 of the 5L. For EQ-5D-Y-3L, ‘*sedikit masalah*’ was confirmed as suitable to represent ‘some problems’ (level 2 of the 3L). However, for EQ-5D-Y-5L, ‘*sedikit masalah*’ was considered more suitable in representing ‘a little bit of problems’ (level 2 of the 5L). From a translation perspective, it appears that the adjacent severity labels influence the interpretation of the word**s**, perhaps related to response spreading [39]. This means that the label for the intermediate level 2 of the 3L can no longer represent the intermediate level of the 5L**.** The logical redistribution of EQ-5D-Y-3L to the EQ-5D-Y-5L Indonesia version was mapped to its wordings. We present the differences between the EQ-5D adult and youth versions, and also between the English and Indonesian versions, in Additional file 2. Equivalent levels in EQ-5D-Y-3L and EQ-5D-Y-5L are connected by a solid arrow (→), whilst different levels still considered as consistent responses are connected by dashed arrows (-->). It is important to note the differences between redistributions:

- In the English version: the adult EQ-5D-3L level 1-2-3 is equivalent to 1-3-5 in EQ-5D-5L. This is not the case in the youth version: level 1-2-3 in EQ-5D-Y-3L is equivalent to 1–3-4 in EQ-5D-Y-5L.
- Due to the language features in Indonesia, level 2 in EQ-5D-Y-3L has the same wording as level 2 in EQ-5D-Y-5L. Hence, level 1-2-3 in EQ-5D-Y-3L is equivalent to 1-2-4 in EQ-5D-Y-5L.

A response pair is defined as inconsistent if the responses differ by 2 or more levels between the 3L and the 5L [14]. The definition is applied to any other level distribution except for level 2 in EQ-5D-Y-3L distributed to level 1 in EQ-5D-Y-5L. Redistribution of responses from ‘slight’ to ‘no problems’ in a more refined system could be considered an error rather than as a possible valid redistribution. Inconsistency can be weighted by the size of the deviation, ranging from 1 (responses differ by 2 levels) to 3 (responses differ by 4 levels) [14].

#### Convergent validity

Convergent validity was tested by correlating the dimension scores of both versions of EQ-5D-Y with related items in the PedsQL Generic Core Scale and the disease-specific instruments. These validity tests assume a monotonic relationship between the scores derived from the generic EQ-5D-Y instrument and the condition-specific measures. The Spearman rank correlation coefficient is interpreted as: absent if r < 0.20, weak if 0.20 < r < 0.35, moderate for 0.35 < r < 0.50, and strong for r > 0.50 [12].

We expected correlations between the EQ-5D-Y mobility dimension to any other items and dimensions related to the physical functions of PedsQL and disease-specific modules. We did not expect correlations with respect to the ‘looking after myself’ and ‘usual activities’ dimensions of EQ-5D-Y since these are not contained in the other questionnaires. The pain dimension of EQ-5D-Y was expected to correlate with the physical and pain-related items in the parallel questionnaires. We also expected a correlation between the worried/sad/unhappy dimension and the items related to feeling (PedsQL) and anxiety (disease-specific modules). The correlations between EQ-5D-Y and the other HRQoL questionnaires were expected to be moderate (0.35 < r < 0.50) and negative.

#### Retest analysis

Test–retest reliability was assessed between the baseline and *t*_*retest*_ in patients who reported no change on their check of change question. Gwet’s AC1 was used to determine a reliability coefficient as it provides better stability than Cohen’s Kappa [40, 41]. Gwet’s AC1 coefficient is less affected by low prevalence found in certain dimensions of our study sample. A Gwet’s AC of < 0.20 was interpreted as poor, 0.21–0.40 as fair, 0.41–0.60 as moderate, 0.61–0.80 as substantial, and > 0.81 as almost perfect agreement [41].

#### Responsiveness

Responsiveness is defined as the ability to capture change over time when change is expected [42]. In our study, responsiveness analysis aimed to report the proportion of aligned changes in EQ-5D-Y levels with the check of change question. Check of changes served as an external criterion to differentiate between patients with changes (improved/deteriorated) and without changes. We reported for each dimension the proportion of patients who gave a lower EQ-5D-Y level (in the improved group), a higher-level (in the deteriorated group), or an equal level (in the stable group).

## Results

### Participants

The characteristics of the study sample are presented in Table 1. 8% of potential participants decided not to participate in the study. The final sample was 286 participants of whom 38% were female, and the mean age was 11.2 (SD = 2.4). 48.3% of patients asked for assistance (for example, in the form of reading the questions aloud and writing down participants’ answers), most of whom were acutely ill (89.8%). The most frequent illnesses were acutely ill (43.4%), major beta-thalassemia (23.7%), haemophilia (18.8%), and AcLL (14%). Drop-out from baseline was 15% for *t*_*retest*_ and 20% for *t*_*followup.*_

**Table 1:** Characteristics of study participants

Description	Major Beta Thalassemia	Acutely ill	Acute Lymphoblastic Leukaemia	Haemophilia	All subjects
Number of patients					
*t*_*baseline*_	68	124	40	54	286
*t*_*retest*_	66	94	39	44	243
*t*_*followup*_	65	90	28	46	229
% female	54.4	43.5	42.5	1.9	38.1
Mean age (SD)	11.6 (2.4)	10.9 (2.3)	10.9 (2.5)	11.9 (2.6)	11.2 (2.4)
Age at first diagnosis	4.5 (3.2)	NA	7.6 (3.7)	3 (3)	NA
Patients eligible for analysis*					
*t*_*retest*_	19	8	6	11	44
*t*_*followup*_	62	88	26	43	219
Hospital visit					
Every week	–			27	
Every 2 weeks	35	NA	NA	20	NA
Every 3 weeks	24			NA	
Every month	8			6	
Other	1			–	

^*^Patients eligible for analysis are the patients who report expected health status at retest (indicate no change) and responsiveness (improved). The large difference between eligible patients for retest and follow-up was because a small proportion of patients reported no changes in their health during the retest assessment

### Feasibility and ceiling effect

There were no missing answers for either EQ-5D-Y-3L or EQ-5D-Y-5L, indicating excellent feasibility for both instruments. There was no significant reduction of the ceiling effect in overall (11111) and on each dimension of EQ-5D-Y-5L compared to EQ-5D-Y-3L (Table 2). At *t*_*followup*_, patients reporting 11111 increased compared to baseline, as the patients returned to normal health.

**Table 2:** Ceiling Effect of EQ-5D-Y-3L and EQ-5D-Y-5L

Time	Instrument	Ceiling effect (%)					
		Patients reporting no problems (%)					
		Mobility	Looking after myself	Usual activities	Pain/ discomfort	Worried/sad/unhappy	Overall (11111)
*t*_*baseline*_	EQ-5D-Y-3L	69.9	62.6	51.0	39.2	65.0	21.3
	EQ-5D-Y-5L	71.7	60.1	48.6	36.0	65.7	17.5
	*p* value*	0.54	0.27	0.36	0.19	0.88	0.88
*t*_*followup*_	EQ-5D-Y-3L	73.8	74.1	66.1	62.9	68.2	53.5
	EQ-5D-Y-5L	74.5	74.8	66.8	60.1	68.9	50.7
	*p* value*	0.63	1.00	1.00	0.09	1.00	1.00

Disease Group	Instrument	Ceiling effect per disease group at baseline (%)					
		Patients reporting no problems (%)					
		Mobility	Looking after myself	Usual activities	Pain/ discomfort	Worried/sad/unhappy	Overall (11111)
Thalassemia	EQ-5D-Y-3L	92.6	75	95.6	70.6	80.9	57.4
	EQ-5D-Y-5L	89.7	79.4	97.1	63.2	75	58
Acutely Ill	EQ-5D-Y-3L	69.1	34.7	34.7	28.2	58.1	13.1
	EQ-5D-Y-5L	71.8	25	29.8	25.8	60.5	18
AcLL	EQ-5D-Y-3L	65	60	50	42.5	47.5	16.4
	EQ-5D-Y-5L	70	57.5	45	40	47.5	16
Haemophilia	EQ-5D-Y-3L	48.1	87	59.3	22.2	74.1	13.1
	EQ-5D-Y-5L	50	85.2	66.7	22.2	79.6	8

*The *p* value was tested using McNemar test with level of confidence interval < 0.05

### Redistribution properties

Table 3 shows the score redistribution from EQ-5D-Y-3L to EQ-5D-Y-5L. Most of the patients who reported 1 on EQ-5D-Y-3L also reported 1 on the EQ-5D-Y-5L version. Patients who reported 2 (some problems) on EQ-5D-Y-3L mostly used level 2 (a little bit of a problem) in EQ-5D-Y-5L in all dimensions. In the mobility, pain/discomfort, and worried/sad/unhappy dimensions, most who reported level 3 (a lot of problems) on EQ-5D-Y-3L redistributed to level 4 (a lot of problems) on EQ-5D-Y-5L. Meanwhile, on the other 2 dimensions, most level 3 responses on EQ-5D-Y-3L were redistributed to level 5 in EQ-5D-Y-5L. Inconsistencies ranged from 8.7% (mobility) to 16.1% (worried/sad/unhappy). The lowest average consistency weight was for mobility and worried/sad/unhappy (1.1), and the highest for usual activities (1.4).

**Table 3:**
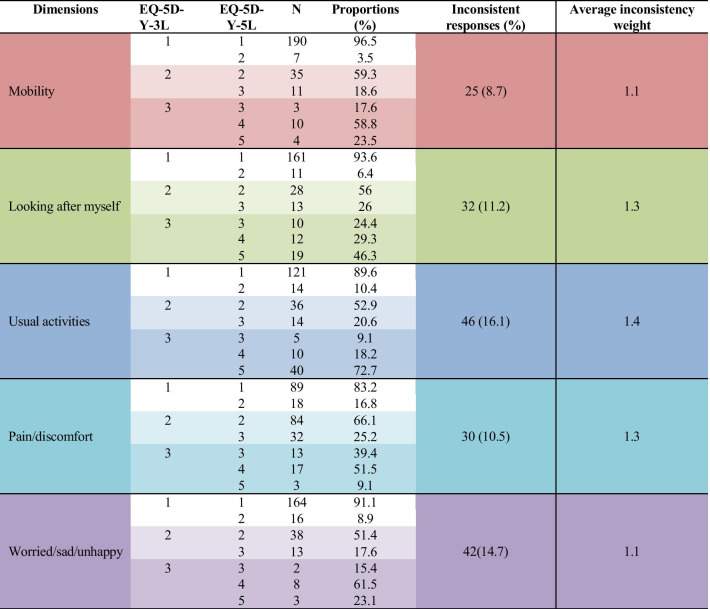
Redistribution properties from EQ-5D-Y-3L to EQ-5D-Y-5L

### Convergent validity of EQ-5D-Y-3L and EQ-5D-Y-5L

The convergent validity analysis employing the PedsQL Generic Core Scales ™ instrument was carried out by disease group. Both versions of EQ-5D-Y had an equal correlation with related items on PedsQL Generic (Table 4). The magnitudes were weak to strong depending upon the variance within dimension**s** in each patient group.

**Table 4:** Convergent Validity of EQ-5D-Y-3L and EQ-5D-Y-5L dimensions with PedsQL Generic Core Scales; Spearman rank correlation

Group	Dimensions	Mobility		Looking After Myself		Usual Activities		Pain/Discomfort		Worried/Sad/Unhappy	
		EQ-5D-Y-3L	EQ-5D-Y-5L	EQ-5D-Y-3L	EQ-5D-Y-5L	EQ-5D-Y-3L	EQ-5D-Y-5L	EQ-5D-Y-3L	EQ-5D-Y-5L	EQ-5D-Y-3L	EQ-5D-Y-5L
Major Beta Thalassemia	PedsQL Total					.41a	.40^a^	.48^a^	.35^a^		
	PedsQL ‘feeling’							.26^b^			
	PedsQL ‘studies’										
	PedsQL Physical	.36^a^	.29^b^					.43^a^	.36^a^		
	PedsQL item 1(hard to walk)	.33^a^	.34^a^								
	PedsQL item 2 (hard to run)	.32^a^	.29^b^								
	PedsQL item 7 (I hurt)	.20^b^						..44^a^	.38^a^		
Acutely Ill	PedsQL Total							.22^b^	.20^b^	.22^b^	.19^b^
	PedsQL ‘feeling’										.20^b^
	PedsQL ‘studies’										
	PedsQL Physical								.23^a^		
	PedsQL item 1(hard to walk)	.25^a^	.24^a^								
	PedsQL item 2 (hard to run)										
	PedsQL item 7 (I hurt)								.24^a^		
Acute Lymphoblastic Leukaemia	PedsQL Total	.68^a^	.71^a^	.65^a^	.61^a^	.57^a^	.45^a^	.70^a^	.57^a^	.47^a^	
	PedsQL ‘feeling’									.45^a^	
	PedsQL ‘studies’										
	PedsQL Physical	.73^a^	.74^a^					.78^a^	.71^a^		
	PedsQL item 1(hard to walk)	.60^a^	.62^a^								
	PedsQL item 2 (hard to run)	.62^a^	.58^a^								
	PedsQL item 7 (I hurt)	.65^a^							.53^a^		
Haemophilia	PedsQL Total	.30^b^		.31^b^	.30^b^	.40^a^		.27^b^			
	PedsQL ‘feeling’										
	PedsQL ‘studies’					.34^b^					
	PedsQL Physical	.34^b^	.27^b^						.30^b^		
	PedsQL item 1(hard to walk)										
	PedsQL item 2 (hard to run)	.35^a^	.33^b^								
	PedsQL item 7 (I hurt)	.35^a^									

^a^Significant at the 0.01 level

^b^Significant at the 0.05 level

Correlation coefficients with *p* value above 0.05 not shown here

Comparable performances appeared in the correlations of EQ-5D-Y-3L and EQ-5D-Y-5L with disease-specific instruments**.** Dimensions with sufficient variance showed at least moderate correlations to related dimensions in EQ-5D-Y (Table 5). EQ-5D-Y-3L and EQ-5D-Y-5L performed about equally.

**Table 5:** Convergent validity of EQ-5D-Y-3L and EQ-5D-Y-5L dimensions with disease specific modules; Spearman rank correlation

Instrument	Dimensions	Mobility		Looking after myself		Usual activities		Pain/discomfort		Worried/Sad/Unhappy	
		EQ-5D-Y 3L	EQ-5D-Y-5L	EQ-5D-Y 3L	EQ-5D-Y-5L	EQ-5D-Y 3L	EQ-5D-Y-5L	EQ-5D-Y 3L	EQ-5D-Y-5L	EQ-5D-Y 3L	EQ-5D-Y-5L
TranQoL	Physical	0.25^b^	0.30^b^			0.38^a^	0.43^a^	0.42^a^	0.36^a^		
	Emotional					0.27^b^	0.32^a^	0.33^a^	0.24^b^	0.32^a^	
	Family										
	School									0.30^a^	
	Total	0.24^b^	0.31^b^			0.37^a^	0.41^a^	0.36^a^		0.31^b^	0.25^b^
HaemoQol (Haemophilia)	Physical Health		0.33^b^		0.33^b^	0.29^b^		0.38^a^	0.40^a^		
	Feeling								0.30^b^		
	Sport	0.30^b^			0.28^b^	0.45^a^		0.32^b^	0.29^b^		
	Dealing										
	Treatment					0.34^b^	0.30^b^				
	Total	0.28^b^		0.28^b^		0.28^b^					
PedsQoL (Cancer)	Pain	0.48^a^	0.41^a^	0.48^a^	0.47^a^	0.45^a^	0.38^b^	0.57^a^	0.61^a^	0.48^a^	0.48^a^
	Nausea	0.39^b^	0.32^a^	0.35^b^	0.33^b^			0.46^a^		0.40^b^	
	Procedure Anxiety					0.34^b^		0.49^a^	0.47^a^		
	Treatment Anxiety							0.44^a^			
	Worry	0.46^a^	0.45^a^	0.39^b^	0.32^b^	0.36^b^	0.33^b^	0.42^a^	0.38^a^	0.51^a^	
	Total	0.55^a^	0.47^a^	0.43^a^	0.38^a^	0.42^a^	0.33^a^	0.66^a^	0.50^a^	0.34^b^	

^a^Significant at the 0.01 level

^b^significant at the 0.05 level

Correlation coefficients with *p* value above 0.05 not shown here

### Test–Retest

There were 44 out of 243 possible pairs (18.1%) where patients indicated no change in their health. EQ-5D-Y-5L showed slightly better stability in all dimensions than EQ-5D-Y-3L, with at least substantial agreement (Gwet’s AC1 coefficient above 0.61) (see Additional file 3).

### Responsiveness

**Fig. 1:**
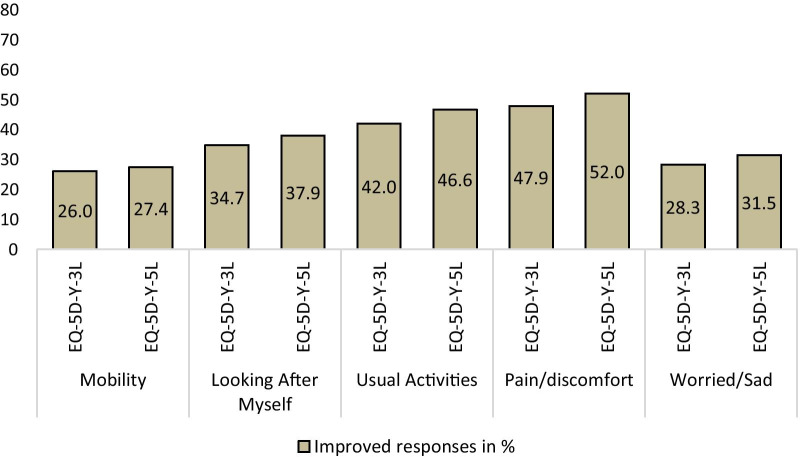
Responsiveness of EQ-5D-Y-3L and EQ-5D-Y-5L

For 229 patients measured in t_followup_, 95.6% indicated their condition had improved, 2.2% stayed the same, and 2.2% deteriorated. Patients with stable and deteriorated conditions were excluded from analysis since the percentages were very low. The proportion of ‘improved’ patients reporting positive changes on EQ-5D-Y-5L dimensions was larger than on EQ-5D-Y-3L dimensions (Fig. 1).

## Discussion

This study compares the performance of EQ-5D-Y-3L with EQ-5D-Y-5L for a broad range of paediatric patients followed over the course of their medical treatment. We did not find any sign that the increased number of response levels jeopardized the feasibility or the validity of the instrument. The EQ-5D-Y-3L and EQ-5D-Y-5L instruments were close in terms of ceiling and convergent validity. EQ-5D-Y-5L was slightly better in terms of reliability and responsiveness. Our results demonstrated that extending the number of levels might not necessarily give ‘superiority’ to EQ-5D-Y-5L over EQ-5D-Y-3L. In this sample and using the current analysis, most of the benefits of increasing the response levels appear to be that EQ-5D-Y-5L performance was better in monitoring health changes over time.

Closer inspection of the redistribution tables reveals some evidence that the ‘accuracy’ of EQ-5D-Y-5L is better than that of EQ-5D-Y-3L. Patients who responded 3 (‘a lot of problems’) on EQ-5D-Y-3L tended to distribute their answers not only to level 4 (‘a lot of problems’), but also to levels 3 and 5 in EQ-5D-Y-5L. Even for ‘looking after myself’ and ‘usual activities’, level 3 in EQ-5D-Y-3L was distributed mostly to level 5 (cannot). This is an indication that the endpoint in EQ-5D-Y-3L was interpreted as a milder condition than the endpoint in EQ-5D-Y-5L. In other words, the EQ-5D-Y-3L version did not cover the whole spectrum of severity that a patient might have had, and the extended range of EQ-5D-Y-5L improved the measurement of severe health states.

We did not find a significant reduction in the ceiling effect. This can be explained in that the Indonesian translation does not ‘insert’ a new level between the top level and the second level. Thus any reduction of the ceiling effects should have come from response spreading. It could be, in children, that the semantic labels of the levels powerfully reduced any effects of response spreading. It could also be that respondents validly ticked the ‘non problem’ level, as they perceived no additional need for care. Indeed, the insertion of an additional level between the top and the second level in the adult version of EQ-5D reduced, but did not eliminate the celling effect (from 20.2% 3L to 16.0% 5L) [13]. This all suggests that the so-called ‘ceiling effect’ of EQ-5D might be more of a real phenomenon, and not necessarily result from any deficiency in the questionnaire.

The inconsistencies were higher than reported in other studies in adults [10, 12, 13, 43] and children [15]. There are two possible reasons: first, ordering of the questionnaires. By presenting EQ-5D-Y-5L first, we anticipated that ‘in-between level’ avoidance might appear stronger in our young and sick population. However, this decision apparently led to another limitation, namely inconsistencies in participants' responses. The presentation of disease-specific modules between administrations of EQ-5D-Y-5L and EQ-5D-Y-3L may have changed how patients perceived their health. Second, the number of inconsistencies might be related to the age of the patient. Nearly fifty percent of patients who gave inconsistent responses were below 10 years old. Their cognitive capacity might explain these inconsistencies.

The convergent validity of both versions of EQ-5D-Y with the PedsQL Generic Core Scales instrument spread from weak to strong. The low correlation**s** might be related to the limited variance captured by generic measurements such as EQ-5D-Y and PedsQL Generic Core Scales. For instance, if the mental dimensions have not been affected, then the maximal variance of scores cannot be reached. Indeed, both versions of EQ-5D-Y showed stronger correlations with the disease-specific instruments that focused on those dimensions most likely to be affected, reducing ‘unused potential variance’. Several coefficients observed were below the expected correlations with EQ-5D-Y. As an example, physical dimensions in TranQol and HaemoQol had weak correlations with mobility in both versions of EQ-5D-Y. The same applied to the emotional dimension of the two instruments with respect to the worried/sad/unhappy dimension of EQ-5D-Y. Inspecting the items, it can be observed that not all items corresponded closely with the expected dimension in EQ-5D-Y. For example, one of the items in the physical dimension of TranQol is: *‘I was able to participate in as many social events as I wanted to*’. This item was not related to the mobility dimension in EQ-5D-Y. The differences explain the weak correlations between several dimensions in the disease-specific module**s** and the EQ-5D-Y dimensions.

Indonesian language features play a role in the translation of the descriptive system in EQ-5D-Y. As mentioned earlier in the methods section, level 2 of EQ-5D-Y-3L was equal to level 2 instead of level 3 in EQ-5D-Y-5L. We believe this language specificity did not restrain the transferability of our findings into other settings. Since we followed the translation protocol and worked together with the Version Management Committee of the EuroQol office, the instrument wordings were considered to be equal to the other language versions of EQ-5D-Y.

There are three potential weaknesses of this study that need attention. The first is related to the limited scope of feasibility assessed in this study: missing responses. Feasibility should be evaluated further by employing several indicators, e.g., completion time, qualitative assessment, and participant preferences. Future EQ-5D-Y studies might aim to include such indicators. Second, it is worth considering the different recall periods in EQ-5D-Y and the PedsQL Generic Core Scale. While EQ-5D-Y asks for the patient’s health 'today', the PedsQL asks for the patient’s health during the 'last month'. The reference period could have affected patients’ responses, especially in the acutely-ill children, and could explain the low correlation between the two instruments. We were aware that the acute version of PedsQL with a shorter recall period (7 days) was available [44, 45], but the disease-specific modules were not. Having two different time frames (today and 1 month) in one set of questionnaires was considered to be a better strategy than having three (today, 7 days, and a month). The limited study published from the PedsQL acute version was also another consideration in not using this version in our study. Third, we could not compare the responsiveness for EQ-5D-Y-3L vs. EQ-5D-Y-5L using values from the general public, which are often referred to as ‘utilities’. This was because a ‘youth tariff’ (or utilities) for the Indonesian child population was not available. Future studies could consider expanding the level range for the check of changes question from 3 to 7 and correlating these changes to population utility scores. However, we would expect results resembling those reported by Janssen, Bonsel, Luo [11] in their comparison study of EQ-5D-3L and EQ-5D-5L in adults, in view of the similar psychometric evidence for the youth and adult versions with respect to their different features.

## Conclusion

The EQ-5D-Y-5L instrument performs slightly better than the simpler 3L version in terms of stability (test–retest) and responsiveness performance and accuracy, especially for severe states. The supposed ceiling effect is not much different between the versions. Moreover, we could not find any signs that the increased number of answer levels makes the questionnaire less applicable, or less valid, in children. Our conclusion therefore is that the increase in the number of levels of EQ-5D-Y from 3 to 5 comes with small improvements in psychometric performance without jeopardizing validity for patients with low or immature cognitive capacities such as children.

## Supplementary Information

**Additional file 1**. Data Collection Time Frame.

**Additional file 2**. Redistribution Score of EQ-5D.

**Additional file 3**. Test-retest Reliability.

## Data Availability

Data from the present study belongs to the authors. Any request to access the data can be sent to the corresponding author.

## Abbreviations

HRQoL: Health-Related Quality of Life
QALYs: Quality Adjusted Life Years
AcLL: Acute Lymphoblastic Leukaemia
SD: Standard Deviation
