# Supplementary figures and images for: Comparing measurement properties of EQ-5D-Y-3L and EQ-5D-Y-5L in paediatric patients
Source: Health Qual Life Outcomes. 2021 Nov 15;19:256. doi: 10.1186/s12955-021-01889-4 (PMC8591892; doi:10.1186/s12955-021-01889-4)

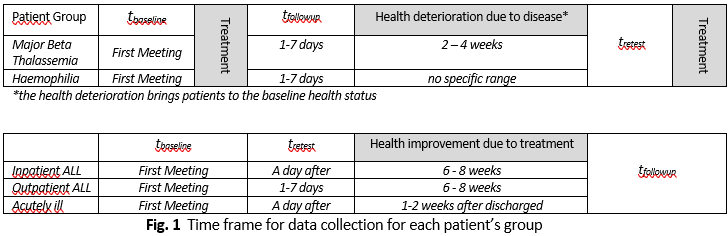

Supplement: Supplementary file 1 — Additional file 1. Data Collection Time Frame. [file 12955_2021_1889_MOESM1_ESM.docx]
