# Supplementary material for: Comparing measurement properties of EQ-5D-Y-3L and EQ-5D-Y-5L in paediatric patients
Source: Health Qual Life Outcomes. 2021 Nov 15;19:256. doi: 10.1186/s12955-021-01889-4 (PMC8591892; doi:10.1186/s12955-021-01889-4)
Supplement: Supplementary file 2 — Additional file 2. Redistribution Score of EQ-5D. [file 12955_2021_1889_MOESM2_ESM.docx]

| **Redistribution of EQ-5D Youth Versions** | | | | | | | | | | |
| --- | --- | --- | --- | --- | --- | --- | --- | --- | --- | --- |
|  | | | | | | | | | | |
| EQ-5D-Y-3L and EQ-5D-Y-5L (English Version) | | | | |  | EQ-5D-Y-3L and EQ-5D-Y-5L (Indonesian Version) | | | | |
| **EQ-5D-Y-3L** | **Level** |  | **Level** | **EQ-5D-Y-5L** |  | **EQ-5D-Y-3L** | **Level** |  | **Level** | **EQ-5D-Y-5L** |
| no problems | 1 |  | 1 | no problems |  | tidak kesulitan | 1 |  | 1 | tidak kesulitan |
| some problems | 2 |  | 2 | a little bit of problems |  | sedikit kesulitan | 2 |  | 2 | sedikit kesulitan |
|  |  |  | 3 | some problems |  |  |  |  | 3 | cukup kesulitan |
| a lot of problems | 3 |  | 4 | a lot of problems |  | sangat kesulitan | 3 |  | 4 | sangat kesulitan |
|  |  |  | 5 | cannot/extreme |  |  |  |  | 5 | tidak bisa/amat sangat |

| **Redistribution of EQ-5D Adult Versions** | | | | | | | | | | |
| --- | --- | --- | --- | --- | --- | --- | --- | --- | --- | --- |
|  | | | | | | | | | | |
| EQ-5D-3L and EQ-5D-5L (English Version) | | | | |  | EQ-5D-3L and EQ-5D-5L (Indonesian Version) | | | | |
| **EQ-5D-3L** | **Level** |  | **Level** | **EQ-5D-5L** |  | **EQ-5D-3L** | **Level** |  | **Level** | **EQ-5D-5L** |
| no problems | 1 |  | 1 | no problems |  | tidak kesulitan | 1 |  | 1 | tidak kesulitan |
| some problems | 2 |  | 2 | slight problems |  | mempunyai kesulitan | 2 |  | 2 | sedikit kesulitan |
|  |  |  | 3 | moderate problems |  |  |  |  | 3 | cukup kesulitan |
| unable/extreme | 3 |  | 4 | severe problems |  | tidak bisa/amat sangat | 3 |  | 4 | sangat kesulitan |
|  |  |  | 5 | unable/extreme |  |  |  |  | 5 | tidak bisa/amat sangat |

|  | : Equivalent level |
| --- | --- |
|  | : Different level but considered as consistent |

**Fig. A2 Comparison of redistribution between EQ-5D-Y-3L to EQ-5D-Y-5L and redistribution of EQ-5D-3L to EQ-5D-5L (English and Indonesian versions)**
