# Supplementary material for: Comparing measurement properties of EQ-5D-Y-3L and EQ-5D-Y-5L in paediatric patients
Source: Health Qual Life Outcomes. 2021 Nov 15;19:256. doi: 10.1186/s12955-021-01889-4 (PMC8591892; doi:10.1186/s12955-021-01889-4)
Supplement: Supplementary file 3 — Additional file 3. Test-retest Reliability. [file 12955_2021_1889_MOESM3_ESM.docx]

| **Dimensions** | **EQ-5D-Y-3L** | | **EQ-5D-Y-5L** | |
| --- | --- | --- | --- | --- |
|  | **Percentage Agreement** | **Gwet’s AC** | **Percentage Agreement** | **Gwet’s AC** |
| Mobility | 89.5 | 0.84 | 92.0 | 0.88 |
| Looking after myself | 92.4 | 0.89 | 96.0 | 0.94 |
| Usual activities | 85.2 | 0.74 | 87.5 | 0.78 |
| Pain/discomfort | 87.5 | 0.76 | 89.2 | 0.80 |
| Worried/sad/unhappy | 80.7 | 0.69 | 86.4 | 0.78 |

**Table A3 -- Test-retest Reliability of EQ-5D-Y-3L and** **EQ-5D-Y-5L**
